# Antigenic Architecture of the H7N2 Influenza Virus Hemagglutinin Belonging to the North American Lineage

**DOI:** 10.3390/ijms25010212

**Published:** 2023-12-22

**Authors:** Aleksandr V. Lyashko, Tatiana A. Timofeeva, Irina A. Rudneva, Natalia F. Lomakina, Anastasia A. Treshchalina, Alexandra S. Gambaryan, Evgenii V. Sorokin, Tatiana R. Tsareva, Simone E. Adams, Alexey G. Prilipov, Galina K. Sadykova, Boris I. Timofeev, Denis Y. Logunov, Alexander L. Gintsburg

**Affiliations:** 1 The Gamaleya National Research Center for Epidemiology and Microbiology, Ministry of Health of the Russian Federation, 123098 Moscow, Russiatimofeeva.tatyana@inbox.ru (T.A.T.);; 2 Federal Scientific Center for the Research and Development of Immune-and-Biological Products, 108819 Moscow, Russiaal.gambaryan@gmail.com (A.S.G.); 3 The Smorodintsev Research Institute of Influenza, the Ministry of Health of the Russian Federation, 197376 St. Petersburg, Russia; 4 Institute of Microbiology, Lausanne University Hospital, 1011 Lausanne, Switzerland

**Keywords:** H7N2 influenza virus, escape mutant, hemagglutinin, antigenic variation, monoclonal antibodies, antigenic mapping

## Abstract

The North American low pathogenic H7N2 avian influenza A viruses, which lack the 220-loop in the hemagglutinin (HA), possess dual receptor specificity for avian- and human-like receptors. The purpose of this work was to determine which amino acid substitutions in HA affect viral antigenic and phenotypic properties that may be important for virus evolution. By obtaining escape mutants under the immune pressure of treatment with monoclonal antibodies, antigenically important amino acids were determined to be at positions 125, 135, 157, 160, 198, 200, and 275 (H3 numbering). These positions, except 125 and 275, surround the receptor binding site. The substitutions A135S and A135T led to the appearance of an N-glycosylation site at 133N, which reduced affinity for the avian-like receptor analog and weakened binding with tested monoclonal antibodies. Additionally, the A135S substitution is associated with the adaptation of avian viruses to mammals (cat, human, or mouse). The mutation A160V decreased virulence in mice and increased affinity for the human-type receptor analog. Conversely, substitution G198E, in combination with 157N or 160E, displayed reduced affinity for the human-type receptor analog.

## 1. Introduction

Avian flu is a disease caused by influenza viruses where the natural hosts are birds. Clinical signs of the disease depend on viral properties, as well as on the species infected and immune status of its host [1]. Many representatives of the H5 and H7 influenza virus subtypes are very dangerous due to their high pathogenicity and mortality, which can reach 100%.

The H7 low pathogenic avian influenza viruses circulate among wild birds, mainly waterfowl [2,3]. Most of these viruses successfully infect poultry, and after a short circulation in a new host, (predominantly *Galliformes*, *gallinaceous species*) can mutate into highly pathogenic variants [4,5,6,7]. Both low and highly pathogenic H7 influenza viruses have repeatedly caused epizootics among poultry, causing significant economic damage. Additionally, people can be infected during close contact with the sick birds [8,9].

Single cases of human infection with H7 influenza viruses have been reported in different countries during last two centuries, such as H7N7 in the USA (1959, 1979), Australia (1977), UK, (1996) and Italy (2013); H7N2 in the USA (2002, 2003, 2016) and UK (2007); H7N3 in Canada (2004), UK (2006) and Mexico (2012); and H7N4 in China (2018) [9,10]. Widespread human infection with avian influenza resulting in disease with varying severities, including death, were observed in the Netherlands in 2003 (H7N7) and in China between 2013–2018 (H7N9) [9,10]. The last examples illustrate the possibility of interspecies transmission by H7 influenza viruses, which pose a potential threat of a new pandemic [11].

The H7 viruses can also infect mammals other than humans. For example, in 1980 in the USA, influenza viruses were isolated from a seal (H7N7, [12]) and in 2016 from cats (H7N2, [13]). In South Korea, H7N2 viruses were discovered in pigs in 2001 [14].

Hemagglutinins (HAs) of all H7 influenza viruses are divided into two large phylogenetic groups from the eastern and western hemispheres, which each contain their own lineages, clusters, and clades. The viruses of the Eurasian lineage have caused numerous human infections in China between 2013–2018 [15]. On the contrary, only singular cases of human infection have been recorded in the western hemisphere [9]. There are three genetic clusters of H7 influenza viruses in the North American lineage. Clusters I and III are composed of viruses that were isolated from wild waterfowl and domestic birds from 1970–1990 (cluster I) and from 1993 to the present (cluster III). The viruses of cluster II were mainly found circulating in poultry in several North American states (Maryland, Pennsylvania, North Carolina, and Virginia) between 1996–2006 [6]. This cluster is further divided into two clades. Clade II-1 contains isolates from 1994–1996, while clade II-2 consists of isolates from 1996–2006 [16].

The H7N2 viruses of the clade II-2 have a feature which distinguishes them from all other H7 influenza viruses, a deletion of eight amino acid residues in HA. The A/chicken/NJ/294598-12/2004 (H7N2) strain studied in this work belongs to this clade. These H7N2 viruses were first discovered in live bird markets in the USA in 1996 [17], and one strain (A/New York/107/2003(H7N2)), was later isolated from an infected human [18,19]. After 2006, there were no signs of these viruses for the following 10 years, until an outbreak of respiratory infection was recorded in cats in a shelter in New York, NY, USA in 2016. The causative agent was an H7N2 influenza virus that was closely related to the chicken viruses of clade II-2. Additionally, a virus with an identical structure was isolated from a veterinarian who was in a contact with a sick cat [13,20].

The expansion of the host range and adaptation from birds to mammals by viruses of different clades, even within the same genetic lineage, may differ by the mechanism of genetic variability affecting various viral proteins. This paper, however, is focused only on changes in the HA of these viruses.

HA, the main viral surface glycoprotein, carries out multiple necessary functions in the viral life cycle. First, HA induces the production of neutralizing antibodies in the infected organism. It is also responsible for the interaction between the virus and cellular receptors [21] and for the penetration of the viral genome into the cell. During penetration, the fusion of the viral lipid envelope with the cellular endosomal membrane is dependent on the pH of the medium [22,23]. Variability of the antigenic structure of HA affects the spread and evolution of the viruses. Neutralizing antibodies produced in the host after first contact with either the influenza virus or vaccination, deactivate the virus upon repeated encounter, preventing the development of infection.

Due to the high mutational variability of RNA viruses, mutants spontaneously arise in the natural influenza virus population. Therefore, the circulating population is normally heterogenic, containing a minor fraction of mutants which cannot be recognized by neutralizing antibodies. Since these mutants escape the immune system response, they are named escape mutants. These escape mutants promote the further spread of the mutated virus and its evolution.

Some amino acid changes in antigenically significant positions of HA have pleiotropic properties and can affect other phenotypic features such as receptor binding, HA thermal stability, the optimal fusion pH (HA activation), or the virulence. All of these alterations can contribute to the expansion of the viral host range, survival of the virus under new conditions, and may promote its evolution, as was shown for H5 viruses [24,25,26].

Normally, it takes time for the appearance of escape mutants during epidemics, epizootics, or vaccination in nature. However, this process can be modulated in a laboratory by utilizing monoclonal antibodies (MABs) to obtain the escape mutants rapidly. Then, antigenically important positions at the 3D structure model of HA can be mapped and studied.

The purpose of this work was to study the antigenic variability of H7N2 influenza viruses in the North American lineage with a deletion in HA (clade II-2). Additionally, the aim was to estimate the influence of this antigenic variability on viral biological properties which may promote viral evolution.

Here, we have obtained escape mutants and studied their phenotypic properties. We have also mapped the antigenically significant amino acid positions in HA to find both specific and common features for different H7 virus lineages of the western and eastern hemispheres.

## 2. Results

### 2.1. Escape Mutants, Their Antigenic Properties and Mutational Variability

The mouse-adapted virus A/chicken/NJ/294598-12MA/2004 (H7N2) (MA/NJ) was obtained by serial passages of the original chicken wild-type A/chicken/NJ/294598-12/2004 (H7N2) (ch/NJ) virus in the lungs of mice [27]. The MA/NJ virus was used as the initial virus to produce escape mutants that have avoided immune pressure and survived in the presence of MABs. MABs used here were 8A3, 9B2, 9B10, 7D11, 7H9, 9E11, and 9G12 [28]. None of the viruses tested here reacted with 8A3, 9B2, and 9B10. The remaining MABs (7D11, 7H9, 9E11, and 9G12) did react with the initial MA/NJ virus, as well as with the non-mouse-adapted wild-type ch/NJ, apart from 7H9. Only some of these MABs also reacted with escape mutants (Table 1). The HA of all studied viruses were sequenced to identify mutations compared to the MA/NJ virus which are responsible for altering the antigenic properties (Table 1 and Table 2).

The HA of the MA/NJ virus has four mutations F125L, D > N157N, G198E, and K328K which differentiate it from the wild-type ch/NJ virus (Table 1 and Table 2). Interestingly, some of the escape mutants, m7H9(1), m7H9(1A), and m7H9(2), had reversions in these four positions. Escape mutant m7H9(1A) had an additional mutation at K327Q. These three mutants and the wild-type ch/NJ virus all have the same profile of reactivity with MABs (Table 1). The mutation at K327Q most likely had little influence on the antigenic reactivity, but the reversions most likely did.

In contrast to all tested viruses and escape mutants, only two mutants, m7D11(2/1) and m7D11(2/6), lost reactivity with MAB 7D11. The loss of reactivity is likely due to a mutation at position 160, as these are the only variants which exhibited a mutation at this location. An additional mutation arose at position 200 in m7D11(2/6), but this replacement did not significantly affect the antigenic reactivity of the virus as reactivity with other MABs remained consistent with m7D11(2/1) (Table 1).

**Figure 1:**
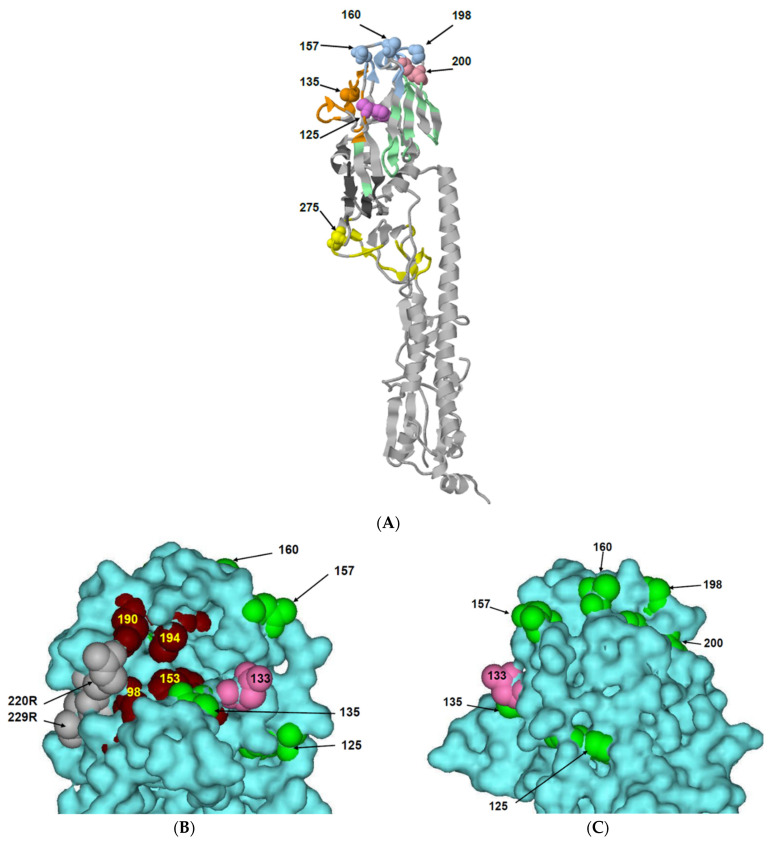
Localization of amino acid changes found in H7N2 escape mutants. The protein structure models for the ribbon (1A) and space filling (1B,C) representations were predicted using the HA of A/environment/New York/30732-1/2005(H7N2) (PDB: 3M5G, 3M5H [18]) and visualized by the program RasMol (http://www.openrasmol.org/). (**A**) The HA monomer with highlighted antigenic sites A (orange), B (blue), C (yellow), and D (green) according to H3 localization and numbering [16]. (**B**,**C**) A molecular model representing the crystal structure of the HA head (blue) containing receptor binding site (RBS, ligand is not shown). Images B and C show rotated views of the same model. Key amino acids of RBS, namely 190E, 194L, 153W and 98Y are shown in brown. Positions which were altered in escape mutants are colored in green. Amino acid 133N which became glycosylated after amino acid substitutions A135S and A135T is shown in pink. Two arginines at positions 220 and 229 (grey) compensate for the deletion of the fragment 221–228 which coincides with the 220-loop in HA.

The comparison of the profile of antigenic reactivity between the initial MA/NJ virus and its escape mutants m9E11(11), m9G12(14), m9G12(17), and m9G12(18A) suggests an importance of the amino acid at position 135. Substitutions A135T or A135S resulted in the appearance of an N-glycosylation site at N133 (Figure 1B,C). Clearly, the appearance of a bulky saccharide structure will disrupt binding potential to the antibodies. Conversely, the substitution D275G in m9G12(18A) did not affect binding to the MABs, which was determined by mutants m9G12(18A) and m9G12(14) exhibiting the same binding profile.

### 2.2. Affinity of Escape Mutants to Cellular Receptor Analogs

Table 1 shows the results of the affinity of escape mutants to the natural glycoprotein fetuin and to 3’SLN and 6’SLN cellular receptor analogues. The 3′SLN and 6′SLN have α2–3 and α2–6 bonds, respectively, between sialic acid and galactose. This corresponds to the avian-type (Sia2–3) and human-type (Sia2–6) cellular receptors.

Influenza viruses with an HA containing a complete 220-loop in the receptor binding site have a pair of amino acids at positions 226 and 228 which are responsible for the recognition of Sia2–3 and Sia2–6 receptors. Probably due to the deletion of a fragment of amino acids, 221–228 in HA/H7, the affinity of wild-type ch/NJ virus for both the Sia2–3 and Sia2–6 receptor analogues differs slightly (Table 1).

Affinity for fetuin conjugate and the Sia2–3 polymer did not change significantly in the mouse-adapted MA/NJ virus carrying substitutions in positions F125L, D157N, G198E, and K328T compared to wild-type, but the affinity for the Sia2–6 polymer was significantly weakened. A decrease in the affinity for the Sia2–6 receptor correlated with the G198E mutation. By increasing the negative charge of HA, the affinity for a negatively charged receptor molecule may weaken. There was also minimal affinity observed in the m7D11(2/6) mutant which has a glutamic acid at both the 198 and 160 positions. Binding to the Sia2–3 receptor is less affected by a change in the charge of amino acids in these positions, probably due to a stronger, specific interaction occurring in the receptor-binding site.

These data indicate that there is some connection between N157 and E198 mutations, as all viruses with this combination of mutations possessed a reduced affinity to the human-type receptor analog (Table 1).

### 2.3. Virulence in Mice, Thermostability, and Optimal pH of Fusion for HA of Escape Mutants

Although multiple mutations arose in the escape mutants, the temperature of HA inactivation did not change compared to the original ch/NJ and MA/NJ viruses. 

In addition to the mutations acquired in HA during the initial adaptation of the chicken influenza virus ch/NJ to mice, the MA/NJ virus acquired one in PB2, E627K [27]. All of the escape mutants characterized here have retained this mutation in PB2. This adaptive mutation has been previously shown to be host species-specific and is the determinant of pathogenicity [29,30]. Without this mutation, the chicken wild-type virus ch/NJ is apathogenic in mice, but the mouse-adapted MA/NJ virus and all escape mutants are pathogenic with a varying amount of virulence from 3.80 ± 0.15 to 5.87 ± 0.25 (Table 2).

A decrease in the pH of fusion by 0.5 units relative to MA/NJ was observed in mutants m9G12(14) and m9G12(18A) carrying the A135T mutation in HA (Table 2). These strains also have the highest virulence among the escape mutants. There was an increase in the pH of fusion for the strain m7D11(2/1) which contained the A160V mutation in HA. This was accompanied by a reduced virulence in mice.

The analysis of the virulence of these escape mutants suggests that the observed increase in virulence for mice of the MA/NJ variant compared to wild-type ch/NJ is dependent not only on the mutation E627K in PB2, but also on substitutions in HA, as the mutants with reversions back to wild-type (L125F, N157D, E198G, T328K) are slightly less virulent than original MA/NJ virus (Table 2).

### 2.4. Mapping Mutations Found in Escape Mutants

To determine which antigenic sites contain the mutations we found in the escape mutants, a 3D structure of a related H7N2 influenza virus of the North American line containing the deletion in HA was used [18]. The location of conformational antigenic sites on the 3D HA/H7 molecule (Figure 1) were established using an analogous HA map from subtype H3 [16]. Mutations at positions 125 and 135 are part of antigenic site A, while the 157, 160, and 198 positions belong to antigenic site B. Position 200 is located on the border between the B and D sites and position 275 is located in the antigenic site C.

**Figure 2:**
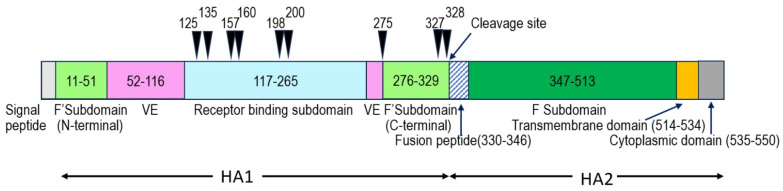
The location of HA amino acid mutations found in the H7N2 influenza virus escape mutants. The HA functional domains are highlighted (H3 numbering) as follows: F’Subdomain—N-terminal and C-terminal Fusion subdomain in HA1 subunit; F Subdomain—Fusion subdomain in HA2 subunit; VE—vestigial esterase domain, black arrows—point mutations. Figure is adapted from [31].

All mutations are also situated in the definite functional domains (Figure 2) according to [18]. Most of them (125, 135, 157, 160, 198, and 200) are in the receptor binding domain (Figure 1B,C and Figure 2). Two mutations (327 and 328) are near the HA cleavage site at position −3 and −2. The mutation at position 275 coincides with the end of the vestigial esterase domain [31] neighboring the C-terminal fusion domain of HA1.

## 3. Discussion

Low pathogenic H7N2 avian influenza viruses with a deletion in the 220-loop of HA arose spontaneously during normal circulation among poultry (mainly chickens) in North America. These viruses gained a foothold in nature and were classified into an independent clade (II-2) of the North American lineage [16,17]. To assess the pandemic potential of viruses lacking the HA 220-loop, we created a mouse-adapted variant using a representative virus of this line called ch/NJ [27]. The mouse-adapted virus variant (MA/NJ) was then used to obtain escape mutants under selection pressure by MABs [28]. The MABs used were produced previously by utilizing a virus of the heterologous Eurasian lineage, A/mallard/Netherlands/12/2000 (H7N3), which was isolated from a wild duck in the Netherlands.

A discordance between the phylogenetic lineages of the virus type used here and that which was used to create the MABs explains why there is non-reactivity between some viruses in this study (North American H7N2) and some of the MABs (H7 Eurasian lineage), as described above (See Section 2.1).

Even so, this approach allowed identification of common antigenically significant positions in HA for different phylogenetic lineages and promoted the discovery of new positions not previously described. For the first time, significant mutations were found at positions 125, 198, and 275 coinciding to antigenic sites A, B, and C (Table 3).

To elucidate potential pleiotropic effects of these mutations, we have studied not only antigenic, but also biological properties of the H7N2 escape mutants. The H7 influenza virus subtype differs from other influenza viruses by features in the structure of their HA receptor-binding site (RBS). All virus subtypes have an RBS that has a broad, shallow pocket located in the top of the head domain and is framed by three structural elements: the 130-loop (a.a. 134–138), 190-helix (188–194), and 220-loop (221–228). Only three subtypes (H7, H10, and H15) also have a 150-loop (154–161), which is formed due to the insertion of two additional amino acid residues between 157 and 158 (H3 numbering) [32,33]. This protruding 150-loop is directed towards the receptor pocket [18,32,34]. Therefore, mutations in this loop may affect affinity to the receptor [35,36]. For example, an H7N9 virus, which contained a T160A mutation, was isolated from humans in China and displayed an enhanced affinity to the human-type Sia2–6 receptor [36].

Another feature of the North American H7N2 clade II-2 viruses is a lack of the 220-loop, which normally has an important role in the RBS of other subtypes. Mutations in the 220-loop of subtypes H1N1 (E190D/G225D) and H2N2/H3N2 (Q226L and G228S) resulted in an adaptation of the viruses to humans and the formation of pandemic strains [18,21]. Despite the absence of the 220-loop, the North American H7N2 viruses save functionality due to a pair of neighboring arginine residues at 220 and 229. These residues compensate for the 221–228 amino acid deletion by maintaining HA structural stability and the ability to bind to the receptor [18] (Figure 1B,C).

Deletions of different lengths in the 220-loop were also observed among other avian influenza virus subtypes. Experimentally derived H9N2 mutants possessed reduced resistance to pH and high temperature and were slightly attenuated relative to the wild-type, which does not harbor the deletion. Additionally, these mutant viruses had dual receptor specificity, able to bind to both avian and human receptors [37]. These phenotypic changes in H9N2 mutants are similar to the properties of the H7N2 escape mutants obtained here (Table 1 and Table 2).

Five mutations found in the H7N2 escape mutants are located in or near the above-mentioned structural elements which frame the RBS, the 130-loop (aa 135), 150-loop (aa 157 and 160), and near the 190-loop (aa 198 and 200) (Figure 1B,C). One mutation, F125L, which occurred during the adaptation of the original avian chicken virus (ch/NJ) to mice, displayed heterogeneity (L > F) with a predominance of 125L in MA/NJ. The other viruses tested here have unambiguity in this position, but some escape mutants (m7H9(1), m7H9(1A), and m7D11(2/6)), have 125F, as does the wild-type virus ch/NJ. The remaining mutants retained 125L (Table 1 and Table 2).

According to the Influenza Research Database (IRD) [38], 125L was found in 13 natural H7 isolates from wild and domestic birds. Among them, only two strains are from North America (A/chicken/New York/Sg-00307/1998(H7N2) and A/American green-winged teal/Illinois/10OS4014/2010 (H7N3)), the others are from the eastern hemisphere. We have not found any previous studies mentioning mutations at position 125 in the literature. This rare F125L mutation is located in the experimentally determined short 117–127 linear epitope (according to IRD data, H3 numbering), which is included in the antigenic site A (Figure 1). The rare occurrence of 125L among natural isolates indicates that viruses with this mutation do not have an advantage in evolution.

Escape mutants with mutations L125F, N157D, E198G and T328K (m7H9(1), m7H9(1A) and m7H9(2)) were obtained using MAB 7H9. As these mutations coincide with ch/NJ, they may be considered as a reversion to wild-type. However, some of the other mutants retained amino acid residues coinciding with the mouse-adapted MA/NJ strain at these same residues, indicating a pressure from MAB 7H9 for particular amino acids at these positions.

The wild-type ch/NJ strain has polymorphism at position 157, where 157D is the dominant and 157N is the minor amino acid residue (Table 1, Table 2 and Table 3). All other escape mutants have unambiguity at this position, which is located in the 150-loop (154–161) near the RBS [18,33,34] and coincides with the short linear epitope (148–163) of antigenic site B (Figure 1) [16,39]. According to IRD data, a variant of the epitope VT-2 (VT-2—type of variant and its number according to IRD, YAEMKWLLS**N**SDNAAFPQ) which has a structure similar to MA/NJ (157N), is quite widespread in natural isolates. In contrast, only one isolate (A/blue-winged teal/Louisiana/AI11-2911/2011 (H7N3)) was discovered with the epitope variant VT-47 (YAEMKWLLS**D**SDNAAFPQ) and has a structure which includes 157D, making it more similar to the wild-type ch/NJ and the escape mutants m7H9(1) and m7H9(1A). This indicates that mutations at 157 are rare in nature.

It is noteworthy that the epitope from 148–163 of the antigenic site B includes another position where mutations A160E and A160V occurred in escape mutants m7D11(2/6) and m7D11(2/1), respectively. No natural isolates were found that have epitopes with the combination of amino acids 157N + 160E, such as the escape mutants 7D11(1) and 7D11(2/6). However, the epitope variant (VT-21) which has 157N + 160V—as does the escape mutant 7D11(2/1)—is present in seven natural isolates from H7N6, H7N3 and H7N1 viruses isolated from wild waterfowl in the USA.

H7 viruses isolated from both birds and humans from the eastern and western hemispheres have the polymorphism A/T/V at amino acid 160 (Table 3) [36,40,41]. By studying the genetic variability of H7N3 viruses during epizootics in Italian poultry farms (2002–2004), where vaccination with a heterologous vaccine was carried out, it was found that the A160T mutation is susceptible to positive selection under vaccine pressure [40].

**Table 3:** Amino acid mutations found in the HA of H7 influenza viruses, the features of these mutations or their effect on viral phenotypic properties.

H3 ^a^	H7 ^b^	Amino Acid ^c^	Ag-Site ^d^	Functional Site ^e^	Feature or Associated Phenotypic Changes	Reference ^f^	
						WH	EH
**125**	115	L, F	**A**	RBD	short linear epitope (117–127, IRD)	this paper	
**135**	125	A, S, T	**A**	RBD, 130-loop	135S—decreased virulence in mice, 135T—increased virulence in mice, A135 T/S—weaker affinity for avian-like receptor analog, 133N+ A135 T/S—133N-glycosylation, changed antigenic reactivity with MABs	this paper	
		S			increased replication and wider tissue distribution, host adaptation to mammals (from poultry to cat or human)	[13]	[8]
		A, T, V		epitope	host adaptation (from wild to domestic birds) to antibody binding sites	[16]	
				epitope	human antibody binding sites		[42,43]
		A→T			HP phenotype in avian viruses	[44]	[44]
		A→T/V			HP phenotype in human H7N7 (the Netherlands) and H7N9 (China) viruses		[15,34,45]
		A→T			at position 133 potential N-linked glycosylation sites (N-X-T/S), increased affinity to avian-type receptor		[34]
**157**	146	N, D	**B**	RBD, 150-loop epitope	short linear epitope (148-163, IRD) N157 + E198—reduced affinity for human-type receptor analog	this paper	
				epitope	human antibody binding sites		[41,42]
**160**	151	A, E, V	**B**	RBD, 150-loop epitope	160V-reduced virulence in mice, increased affinity for human-type receptor analog, 160E + 198E–reduced affinity for human-type receptor analog, short linear epitope	this paper	
		T→A			H7N9, increased binding to human-type influenza receptor, transmission from poultry to human		[46]
					H7N9 human antibody binding epitope		[41]
		A, T		epitope	avian virus vaccine epitope		[36,40,47]
		A, T, V			antigenic diversity in wild bird viruses	[16]	[47]
**198**	189	E, G	**B**	RBD, near 190-loop	epitope (escape mutant), H7N9 viral epitope for human blood antibody	this paper	[41,48]
		E			160E + 198E decrease affinity for the Sia 2–6 receptor due to increase in the HA negative charge	this paper	
**200**	191	K, N	**B/D**	RBD, near 190-loop	H7N2 escape mutant	this paper	
**200**	191	K			B cell epitope in H7N9 HA (human blood antibody)		[49]
**275**	265	D, G	**C**	VED/F ^g^	H7N2 escape mutant, 275G- rare mutation	this paper	
**327/-3**	319	K, Q	**-**	Cleavage site	H7N2 escape mutant	this paper	
		K→R		Cleavage site	HP viruses of poultry (North American H7 cluster III)		[6]
**328/-2**	320	T, K	**-**	Cleavage site	H7N2 escape mutant	this paper	

Notes: ^a^—H3 numbering, ^b^—H7 numbering according to mature HA protein (AAR02640) of A/Netherlands/219/2003 (H7N7) (AY338459, GenBank), ^c^—amino acid residue at defined position or polymorphism, ^d^—antigenic (Ag) site according to HA/H3 map [16] (https://www.ncbi.nlm.nih.gov/pmc/articles/PMC4746648/bin/srep20688-s2.xls, accessed on 18 December 2023). ^e^—functional domain or site, RBD—receptor binding domain, ^f^—reference for H7 influenza viruses from the western hemisphere (WH) or eastern hemisphere (EH), ^g^—VED/F, the boundary between vestigial esterase domain and fusion subdomain, HP—highly pathogenic.

In H7N9 influenza viruses isolated from humans in China, the amino acid residues 157 [42] and 160 [41] were both found to be part of the epitopes that are recognized by neutralizing antibodies from vaccinated people or from those who recovered from infection. Of note, the human H7N9 viruses carrying the T160A mutation, which is located in the 150-loop of HA, have enhanced affinity with the human-type Sia 2–6 receptors [36]. The 160E mutation described here was found in H7N2 escape mutants and is unique (Table 1). Moreover, the replacement of A160E is accompanied by two additional mutations, L125F and K200N (m7D11(2/6)).

Two mutations, E198G and K200N, appeared to only form with other substitutions in the H7N2 escape mutants as each was accompanied by additional mutations at other amino acid positions. As mentioned before (see Section 2.2), there is a proposed connection between N157 and E198 mutations, located respectively in the 150- and 190-loops of the RBS, affecting the human-type receptor binding (Table 1). A similar connection between mutations in these HA loops was shown for H10N8 influenza viruses [33], where the HA (H10) is a member of the same HA phylogenetic subgroup as H7 and H15 [50].

Although amino acids at positions 198 and 200 do not touch the receptor, they are needed to maintain the 3D structure of the 190-loop (Figure 1). Both the 198 and 200 positions from H7N9 viruses isolated from humans during the epidemic in China (2013–2018), were part of the conformational epitopes that are recognized by B-cell antibodies from vaccinated people or those who recovered from illness (Table 3) [41,48,49].

We did not find any previous studies reporting mutations at amino acid 275. This residue is between 274 and 276 which are included in the antigenic site C (Figure 1). In the literature, only mutations in the neighbor, 276, from North American H7 cluster III viruses [6,16] and human H7N9 virus from China are described [15]. Therefore, we suggest that the D275G substitution is a very rare, immunogenic mutation.

Located at antigenic site A, amino acid 135 (Figure 1 and Figure 2, Table 1 and Table 3) attracted particular attention while studying H7 viruses of both the eastern and western hemispheres. In the eastern hemisphere, H7N9 isolates from China have a polymorphism (A, T, or V) at position 135 [15,43,45]. Following positive selection, the 135T mutation was shown to be associated with a highly pathogenic phenotype [44,45]. Additionally, the H7 human-adapted, highly pathogenic isolates were shown to have either 135T or 135V [15]. The amino acid 135 has also been recognized by neutralizing antibodies from humans and has, under the pressure of selection by MAB or human neutralizing antibodies, induced escape mutants [42,43]. In the western hemisphere, North American wild and domestic bird viruses have also been shown to contain a polymorphism at 135 (A, T, or V) [16].

As with our finding (m9E11(11), Table 1 and Table 3), the A135S mutation was detected in H7N2 viruses isolated from cats, a human (North American clade II-2 lineage) [13] and in H7N7 human viruses transmitted from poultry (Netherlands) [8]. This suggests that the amino acid at position 135 affects adaptation to a new host.

Moreover, the A135S/T substitution leads to the appearance of an additional N-glycosylation site at 133N. According to our data, the acquisition of the 133N glycosylation site by escape mutants of H7N2 lead to a decrease in affinity for avian-type receptors. Escape mutants with the A135S (m9E11(11)) and A135T (m9G12(17)) mutations have a lower affinity for the Sia2-3 avian-type receptors than the mouse-adapted MA/NJ strain (Table 1).

On the contrary, the Eurasian H7N7 viruses (A/Netherlands/219/2003 and its derivatives), which have an N-glycosylation site at 133N due to a substitution at A135T, have increased binding affinity to the avian-type Sia2-3-linked sialosides [34]. For viruses of both subtypes, H7N2 and H7N7, glycosylation of 133N did not affect binding with analogues of the human-type Sia2–6 receptors. This contradiction between H7N2 and H7N7 viruses in their affinity to Sia2–3 receptors can be explained in two ways. Firstly, by the deletion of 220-loop in H7N2 viruses. The H7N7 viruses have the residue 226Q which is located in that 220-loop and is directly involved in connection to the receptor [34]. Secondly, the presence of a glycosyl (carbohydrate) residue near the receptor pocket can create steric difficulties and weaken affinity to the receptor [34,51], which coincides with our results presented here.

The pathogenicity of H7 influenza viruses is mainly determined by the proteolytic cleavage site structure in the HA precursor [52]. The cleavage site of low pathogenic viruses contains only one R residue, while the cleavage site of highly pathogenic viruses contains several of these basic amino acids. Two mutations, K327Q and K328T, were found, respectively, at positions -3 and -2 from the proteolytic cleavage site, at the C-terminal end of the HA1 chain. The K328T was considered as a reversion to wild-type. Both cleavage site structures, EKPK**K**R↓G-like ch/NJ and EKPK**T**R↓G-like MA/NJ, correspond to the apatogenic phenotype [53]. According to IRD data, both structures are found in H7 viruses of different bird species on the American continent. A mutation at the neighboring position, K327Q, did not affect the pathogenic properties of the virus and no viruses with such a structure (KPQKR↓GLF) were found.

## 4. Materials and Methods

### 4.1. Viruses

The wild-type avian influenza virus A/chicken/NJ/294598-12/2004 (H7N2, ch/NJ) was kindly provided for study by Dr. Alexander I. Klimov (Centers for Disease Control and Prevention, Atlanta, GA, USA). The mouse-adapted strain A/chicken/NJ/294598-12MA/2004 (H7N2, MA/NJ) was obtained from wild-type ch/NJ virus by 10 serial passages through mouse lungs [27]. Viruses were propagated for 48 h in the allantoic cavities of 10-day-old embryonated chicken eggs at 37 °C and stored −80 °C until use.

### 4.2. Selection of Escape Mutants with Monoclonal Antibodies

A panel of virus-neutralizing monoclonal antibodies (MABs) specific to the HA of A/mallard/Netherlands/12/2000 (H7N3) strain were produced in the Smorodintsev Research Institute of Influenza, Ministry of Health of the Russian Federation (St. Petersburg, Russia) [28].

Escape mutants were obtained according to the previously described method [54]. Briefly, the mouse-adapted virus A/chicken/NJ/294598-12MA/2004 (H7N2) was diluted to a titer of 100 HAU/mL (hemagglutinating units) and mixed with an equal volume of each specific MAB so that each exceeds the neutralizing titer by 100-fold. The mixture was incubated for 1 h at room temperature, followed by propagation in embryonated chicken eggs at 37 °C for 48 h. Harvested allantoic fluid was tested by the hemagglutination assay with 0.75% chicken erythrocytes in phosphate-buffered saline (PBS). Those with positive results were further tested by the hemagglutination inhibition assay with a panel of MABs specific to the HA of A/mallard/Netherlands/12/2000 (H7N3). A virus variant was considered as an escape mutant if it lost or reduced reactivity with the MAB which was used for its production.

### 4.3. Hemagglutination and Hemagglutination Inhibition (HI)

Both tests were performed by a standard method according to [55]. A plastic U-bottom plate (72 wells, M-09, Sovtech, Russia) was used for the hemagglutination assay. The wells of each row contained 500 μL of two-fold serial dilutions from infective allantoic fluids, diluted in PBS. An equal volume of 0.75% solution of chicken erythrocytes in PBS was then added to each well and incubated at 4 °C for 30 min. Hemagglutination was determined by tilting the plate to observe the presence or absence of diffuse agglutinated erythrocytes. The titration was determined to the highest dilution which gave complete hemagglutination (no pellet) representing 1 HAU (hemagglutination unit) and could be accurately calculated from the range of dilutions.

The similar method was used for the hemagglutination inhibition assay. Each well in a row contained 250 μL of two-fold serial dilutions of clonal MABs in PBS. The dilution 8 HAU of virus in 250 μL was then added to each well and incubated for 1 h at 20 °C. Then, 500 μL of 0.75% erythrocytes in PBS were added to each well and incubated at 4 °C for 30 min. The HI titer was determined to be the highest dilution of MABs which caused complete inhibition of 8 HAU of virus.

A negative control with PBS only and a positive control with serum or MABs for which the titer should be within one dilution of the known titer.

### 4.4. Nucleotide Sequencing

The HA gene structure was determined by Sanger sequencing as described earlier [25]. Sequences of escape mutant HA genes were deposited in GenBank with accession numbers MT379530-MT379539 (Table 2).

Complete genome sequences of A/chicken/NJ/294598-12MA/2004 and A/chicken/NJ/294598-12/2004 coincide with sequences represented in GenBank under accession numbers MN400380-MN400387 and MN400388-MN400395, respectively.

### 4.5. Assessment of the Viral Infectious Titer in Chicken Embryos

The 10-day old chicken embryos were used to determine the viral infectious titer. The embryos were infected with 100 μL of 10-fold serial dilutions of the virus into the allantoic cavity (five embryos with every dilution). After 48 h of incubation at 37 °C, the chicken embryos were kept overnight at 4 °C. Then, the allantoic fluid of each embryo was assessed using the hemagglutination assay to detect virus. The viral titer was calculated as the 50% embryo infectious dose (EID_50_) by the routine method of Reed and Muench [56].

### 4.6. Assessment of the Mouse Lethal Dose

BALB/c mice weighing 8–10 g were infected intranasally with 10-fold serial dilutions of influenza virus (50 μL per mouse) under light ether anesthesia (six animals per dilution). The animals were observed for 14 days to assess their changes in body weight and survival. The lethal dose (LD_50_) was calculated by the Reed and Muench method [56]. Virulence was expressed as log_10_ of EID_50_ in one unit of LD_50_.

### 4.7. Assessment of HA Thermostability

Clarified allantoic viruses, standardized to 128 HA units, were incubated in PBS for 40 min at temperatures ranging from 45 °C to 70 °C in a Mastercycler nexus gradient thermocycler (Eppendorf, Germany). After incubation, viruses were titrated by hemagglutination assay with 0.75% chicken erythrocytes. The temperature which resulted in a decrease of 6 log_2_ units in the hemagglutination titer was taken as the temperature of HA heat inactivation [57].

### 4.8. Determining the pH Optimum of Fusion

Clarified allantoic viruses (250 μL), standardized to 128 HA units in PBS, were mixed with 50 μL of a 2.5% suspension of chicken erythrocytes and incubated on ice for 1 h to allow virus binding. After centrifugation at 100× *g*, pellets were resuspended in 250 μL of 2-(N-morpholino)ethanesulfonic acid buffer at various pH values (from 5.0 to 6.5) and incubated at 37 °C for 1 h. Then, samples were precipitated (100× *g*) and supernatants (170 μL) were transferred to 96-well plates to determine the optical density (at 450 nm) which correlates with the erythrocyte hemolysis induced by virus and cell membrane fusion. The lowest pH value that resulted in the highest optical density was taken as the optimal pH. Reported results are the means of pH of fusion ± SD measured in three replicative experiments [57].

### 4.9. Receptor Binding Specificity of Influenza Viruses

The affinity of influenza viruses to cell receptor analogues and fetuin was determined by ELISA. The sialooligosaccharides Neu5Aca2-3Galß1-4GlcNAcß (3’SLN) and Neu5Aca2-6Galß1-4GlcNAcß (6’SLN) were conjugated with a high-molecular polyacrylamide labeled with biotin [21]. The results were presented as a dissociation constant calculated in sialic acid nanomoles (K_diss_, nmol SA).

### 4.10. Statistical Analysis

Analysis of variance (ANOVA) was used for comparing the values of pH of fusion, temperatures of HA inactivation, and results of the HI assay. A probability value equal to 0.05 (*p* < 0.05) was used to indicate that the data were not a result of chance. The *p*-value was calculated by Microsoft Office Excel software 365.

## 5. Conclusions

By obtaining and analyzing the data found here in comparison with published results of other studies, we conclude that the antigenically important amino acid positions in HA are 125, 135, 157, 160, 198, 200, and 275, which surround the receptor binding site apart from 125 and 275.

Some of these mutations have pleiotropic effects on other phenotypic properties of the viruses, giving them an advantage in adapting to new conditions and promoting their evolution. For example, the A135S and A135T substitutions lead to the appearance of an N-glycosylation site at 133N and weakened their affinity for an avian-like receptor analog. Therefore, we suggest that the amino acid at 135 position is responsible for adaptation to a new host.

Another mutation observed here, A160V, decreased virulence in mice, but it increased affinity for the human-type receptor analog, while substitution G198E in combination with 157N or 160E reduced affinity for a human-type receptor analog.

Despite the phylogenetic differences between H7 viruses of western and eastern hemispheres, there are common antigenically important positions, namely 135, 157, 160, 198, and 200 in HA. This knowledge is important when creating, choosing, and evaluating vaccine candidates, as vaccine efficiency depends on the presence of the correct epitope in both the vaccine and the circulating strains. Indeed, the use of an antigenic map allows the determination of which positions in the amino acid sequence need to be evaluated thoroughly.

## Figures and Tables

**Table 1:** Mutants of A/chicken/NJ/294598-12MA/2004 (H7N2) (MA/NJ) mouse-adapted influenza virus which escaped from immune pressure of monoclonal antibodies: mutations in hemagglutinin (HA) and reactivity with MABs and cell receptor analogs are shown here.

	Amino Acid Positions in HA/H7 (H3 Numbering)									Antigenic Reactivity with MABs *				Affinity to Cell Receptor Analogs, K_diss_ (nmol SA) **		
**Virus, Mutant**	**125**	**135**	**157**	**160**	**198**	**200**	**275**	**327**	**328**	**7D11**	**7H9**	**9E11**	**9G12**	**Fet-HRP**	**3’SLN**	**6’SLN**
**MA/NJ**	L>F	A	N	A	E	K	D	K	T	400	3200	1600	3200	200 ± 100	100 ± 50	500 ± 50
**ch/NJ**	F	A	D>N	A	G	K	D	K	K	3200	<100	800	3200	200 ± 100	100 ± 50	200 ± 50
**m7H9(1)**	F	A	D	A	G	K	D	K	K	3200	<100	800	1600	200 ± 100	100 ± 50	100 ± 50
**m7H9(1A)**	F	A	D	A	G	K	D	Q	K	6400	<100	1600	3200	200 ± 100	100 ± 50	100 ± 50
**m7H9(2)**	F	A	D	A	G	K	D	Q	K	3200	<100	800	3200	n.t.	n.t.	n.t.
**m9E11(11)**	L	S	N	A	E	K	D	K	T	1600	<100	<100	<100	300 ± 100	300 ± 50	500 ± 50
**m9G12(14)**	L	T	N	A	E	K	D	K	T	1600	<100	<100	<100	n.t.	n.t.	n.t.
**m9G12(17)**	L	T	N	A	E	K	D	K	T	1600	<20	<100	<100	200 ± 100	200 ± 50	800 ± 50
**m9G12(18A)**	L	T	N	A	E	K	G	K	T	1600	<100	<100	<100	200 ± 100	200 ± 50	800 ± 50
**m7D11(2/1)**	L	A	N	V	E	K	D	K	T	<100	1600	800	3200	300 ± 100	100 ± 50	300 ± 50
**m7D11(2/6)**	F	A	N	E	E	N	D	K	T	<100	12,800	1600	6400	200 ± 100	400 ± 50	1000 ± 50

Notes: The average values of three independent experiments for each test are presented. *p* ≤ 0.05 compared with the value for MA/NJ virus by one-way ANOVA. Essential amino acid differences from A/chicken/NJ/294598-12MA/2004 (H7N2) virus (MA/NJ) are highlighted in yellow or green. ch/NJ—Wild-type virus A/chicken/NJ/294598-12/2004 (H7N2). * The antigenic reactivity was determined by hemagglutination inhibition (HI) titers using a panel of monoclonal antibodies (MABs) against A/mallard/Netherlands/12/2000 (H7N3) influenza virus. An antigenicity difference is considered significant when there is a four-fold difference. Lost antigenic reactivity is highlighted in purple. ** The results of titration with peroxidase-labeled fetuin conjugate (Fet-HRP) and biotinylated polymers 3’SLN (Neu5Acα2-3Galβ1-4GlcNAcβ) and 6’SLN (6’SLN-Neu5Acα2-6Galβ1-4GlcNAcβ) are presented as a dissociation constant expressed in sialic acid nanomoles. A higher value corresponds to a lower affinity for cellular receptor analogues. n.t. indicates not tested.

**Table 2:** Escape mutants selected from A/chicken/NJ/294598-12MA/2004 (H7N2) virus and effect of amino acid changes on virulence in mice and pH of fusion.

Virus, Mutant	GenBank Accession Number	^a^ Amino Acid Changes in HA	^b,c^ Virulence in Mice	^b^ pH of Fusion
MA/NJ	MT379530	125L>F, 157N, 198E, 328T	4.0 ± 0.2	5.6 ± 0.1
ch/NJ	MN400391	125F, 157D>N, 198G, 328K	>9.6	5.2 ± 0.1
m7H9(1)	MT379534	L125F, N157D, E198G, T328K	5.4 ± 0.2	5.4 ± 0.1
m7H9(1A)	MT379535	L125F, N157D, E198G, K327Q, T328K	n.t.	n.t.
m7H9(2)	^d^ MT379535	L125F, N157D, E198G, K327Q, T328K	5.2 ± 0.2	5.6 ± 0.1
m9E11(11)	MT379538	125L, A135S	4.9 ± 0.2	5.4 ± 0.1
m9G12(14)	^d^ MT379536	125L, A135T	3.8 ± 0.15	5.1 ± 0.1
m9G12(18A)	MT379539	125L, A135T, D275G	4.2 ± 0.15	5.1 ± 0.1
m7D11(2/1)	MT379532	125L, A160V	5.9 ± 0.25	5.7 ± 0.1
m7D11(2/6)	MT379533	L125F, A160E, K200N	4.4 ± 0.25	5.4 ± 0.1

Notes: ^a^ Numbering is given for the mature HA/H3 protein of strain A/Aichi/2/68 according to alignment [18]. ^b^ The average values of three independent experiments are presented. *p* ≤ 0.05 compared with value for MA/NJ virus by one-way ANOVA. ^c^ Pathogenicity for mice is represented as log_10_ of EID_50_ in one unit of LD_50_. A lower value corresponds to a higher pathogenicity. ^d^ HA nucleotide sequence is identical to reference strain. n.t. indicates not tested.

## Data Availability

Data contained within the article.
